# The Kidney-Immune-Brain Axis: The Role of Inflammation in the Pathogenesis and Treatment of Stroke in Chronic Kidney Disease

**DOI:** 10.1161/STROKEAHA.124.047070

**Published:** 2025-03-05

**Authors:** Dearbhla M. Kelly, Eoin M. Kelleher, Peter M. Rothwell

**Affiliations:** Wolfson Centre for the Prevention of Stroke and Dementia, Nuffield Department of Clinical Neurosciences (D.M.K., P.M.R.).; Nuffield Department of Clinical Neurosciences (E.M.K.), University of Oxford, United Kingdom.

**Keywords:** cardiovascular diseases, inflammation, renal dialysis, renal insufficiency, chronic, stroke

## Abstract

Cardiovascular diseases such as stroke are a major cause of morbidity and mortality for patients with chronic kidney disease (CKD). The underlying mechanisms connecting CKD and cardiovascular disease are yet to be fully elucidated, but inflammation is proposed to play an important role based on genetic association studies, studies of inflammatory biomarkers, and clinical trials of anti-inflammatory drug targets. There are multiple sources of both endogenous and exogenous inflammation in CKD, including increased production and decreased clearance of proinflammatory cytokines, oxidative stress, metabolic acidosis, chronic and recurrent infections, dialysis access, changes in adipose tissue metabolism, and disruptions in intestinal microbiota. This review focuses on the mechanisms of inflammation in CKD, dialysis and associated therapies, its proposed impact on stroke pathogenesis and prognosis, and the potential role of anti-inflammatory agents in the prevention and treatment of stroke in patients with CKD.

Chronic kidney disease (CKD) affects 8% to 16% of the population worldwide^1^ and contributes significantly to the global burden of cardiovascular disease (CVD).^2^ In particular, CKD is strongly linked to all manifestations of cerebrovascular disease, including stroke, cerebral small vessel disease, and poststroke dementia.^3–5^ There is a stepwise inverse relationship between kidney function, assessed using the estimated glomerular filtration rate (eGFR), and the risk of incident stroke, with risk increasing by 3-fold for CKD stage 3, 4.1-fold for CKD stage 4, 5.4-fold for CKD stage 5, and 7.1-fold for individuals undergoing dialysis when compared with the general population.^6^ Although these risk associations may be attributable to the clustering of traditional cardiovascular risk factors in this group, more recently there has been a growing focus on the potential role of inflammation in this relationship, particularly as greater evidence emerges from general population studies and trials.^7,8^ In this review, we will explore sources of inflammation in kidney disease, the evidence that exists for inflammatory mediators in the pathway from kidney disease to stroke, prognostic implications of inflammation in stroke, and the efficacy of anti-inflammatory therapies for stroke prevention in this group.

## Data Availability Statement

No new data were created or analyzed in this study. Data sharing is not applicable to this article.

## Kidney’s Role in Immune Homeostasis

The kidney plays an important role in maintaining homeostasis of the immune system by clearing circulating cytokines and bacterial antigens such as lipopolysaccharide. Reducing inflammation and immune cell activation can be achieved through the elimination of proinflammatory cytokines and pathogen-associated molecular patterns.^9^ In addition, dendritic cells and macrophages that are resident in the glomerulus, cortical, and peritubular interstitium help maintain peripheral immune tolerance.^10^ There are 2 main types of macrophages: M1 and M2. The M1 subtype generates proinflammatory signals, whereas the M2 subtype exerts anti-inflammatory effects by releasing cytokines such as IL (interleukin)-10 and TGF-β (transforming growth factor-beta). While the balance between M1 and M2 macrophages is normally carefully regulated, impaired kidney function can result in an increased prevalence of the M1 proinflammatory phenotype.^11^

Furthermore, CKD can lead to decreased clearance and increased production of circulating proinflammatory cytokines such as IL-1, IL-6, IL-18, IFN-γ (interferon-gamma), and TNF-α (tumor necrosis factor-alpha), among others.^12^ The activation of certain resident kidney cells, including mesangial cells, endothelial cells, tubular epithelial cells, and podocytes, results in the production of these proinflammatory chemokines, perpetuating the cycle of chronic inflammation.^9^ Additionally, CKD can promote the activation of enzymes that generate reactive oxygen species, such as nicotinamide adenine dinucleotide phosphate oxidase and xanthine oxidase. The increased oxidative stress within the kidney from these reactive oxygen species further activates NF-κB (nuclear factor-kappa B), a transcription factor that amplifies the inflammatory response. NF-κB plays a central role in coordinating and promoting the production of numerous inflammatory cytokines and mediators, including TNF-α, IL-1β, IL-6, IL-8, and IL-12, as well as chemokines like MCP-1 (monocyte chemoattractant protein-1) and RANTES (regulated on activation, normal T cell expressed and secreted), thus exacerbating the inflammatory environment in CKD.^13^

Together, the decreased clearance of proinflammatory cytokines, increased production of these cytokines by resident kidney cells, and the enhanced oxidative stress and NF-κB activation create a feedback loop that sustains and intensifies chronic inflammation in the kidney.

## Sources of Inflammation in CKD

**Figure 1.**
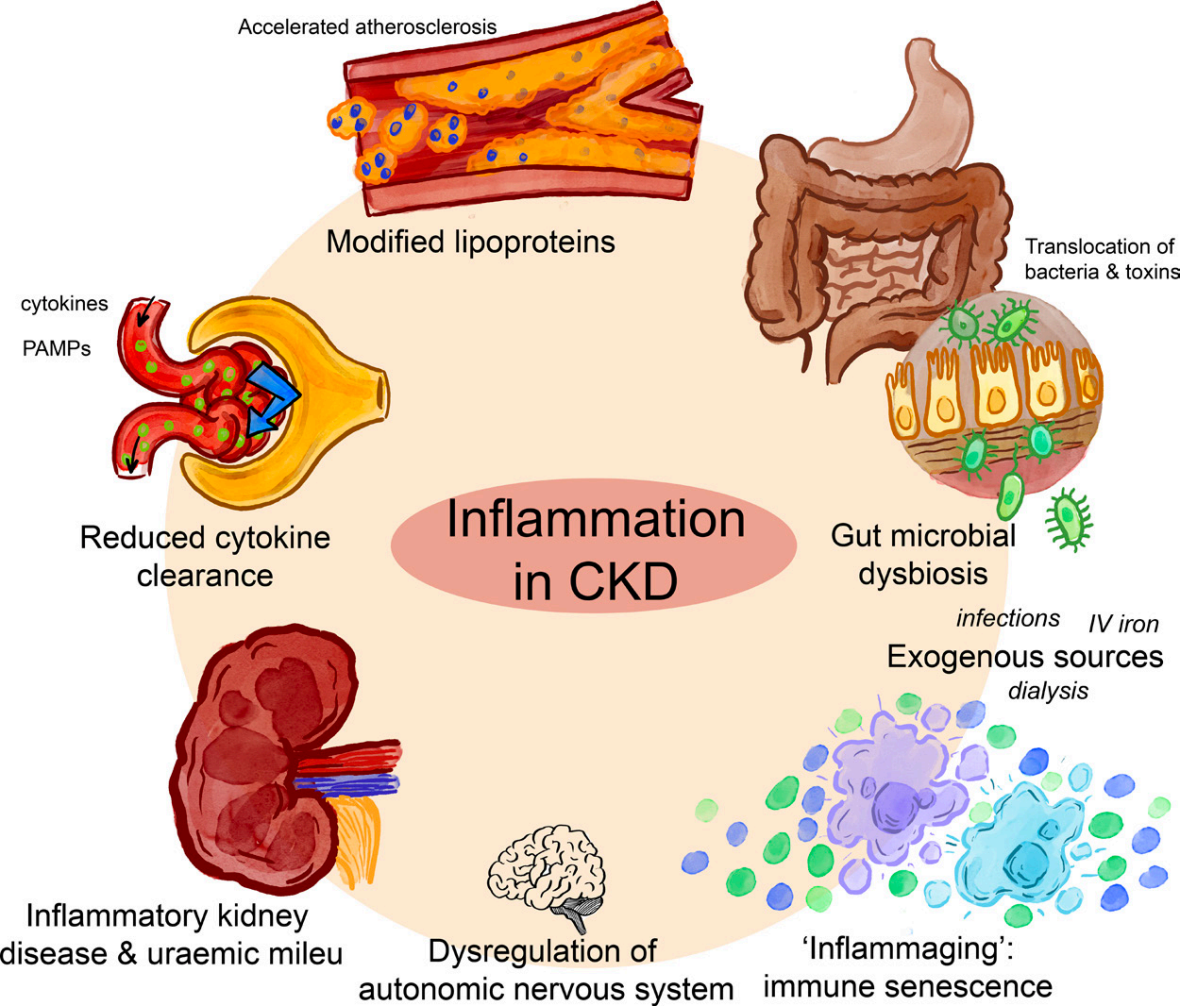
**Sources of inflammation in chronic kidney disease (CKD).** PAMPs indicate pathogen-associated molecular patterns.

Chronic low-grade inflammation is well described in CKD and has been implicated in a myriad of adverse disease outcomes for these patients, including kidney disease progression,^14^ protein-energy wasting,^15^ growth failure in children,^16^ anemia,^17^ all-cause mortality,^18^ as well as CVD.^19^ Several factors contribute to immune dysregulation and inflammatory activation in CKD (Figure 1), including reduced clearance of circulating cytokines, gut microbial dysbiosis, modified lipoproteins, senescent immunology, dysregulation of the autonomic nervous system, and certain kidney disease etiologies.^19^ We will outline the evidence for the importance of these endogenous inflammatory mediators.

### Gut-Derived Bacterial Toxins

In CKD, bacterial toxins can build up due to heightened production, increased absorption resulting from compromised intestinal barrier function, and decreased clearance.^20^ Breakdown of the intestinal epithelial barrier due to loss of tight junction proteins has been described in animal models of CKD^21,22^ and is likely responsible for the increased translocation of gut bacteria and bacterial components into the circulation, subsequently triggering systemic inflammation.^23^ Individuals with CKD exhibit modified gut microbiome compositions, with colonic bacteria generating several uremic toxins including p-cresol sulfate, indoxyl sulfate, and trimethylamine-N-oxide. These uremic toxins produced by the gut microbiota can activate the innate immune system through several mechanisms. For example, endotoxin induces the generation of proinflammatory cytokines by interacting with toll-like receptors located on the cell surface of innate immune effector cells like monocytes or macrophages.^24^ Indoxyl sulfate can enhance the buildup of atherogenic lipoproteins within macrophages by influencing reverse cholesterol transport.^25,26^ Increased circulating levels of these uremic solutes have been associated with elevated levels of inflammatory biomarkers, increased arterial stiffness, and higher cardiovascular morbidity and mortality in patients with kidney disease.^27,28^

### Modified Lipoproteins and Premature Atherosclerosis

CKD promotes proinflammatory lipoproteins by altering their proteome and lipidome through posttranslational protein modifications.^29^ As kidney function deteriorates, urea accumulates and naturally breaks down to produce cyanate, which engages in an irreversible reaction called carbamylation with proteins and free amino groups. Carbamylation of LDL (low-density lipoprotein) enhances its proatherogenic properties, while carbamylation of HDL (high-density lipoprotein) impairs its antiatherogenic functions, collectively promoting atherogenesis and atheroprogression.^30^ Carbamylated LDL also contributes to endothelial dysfunction via inhibition of nitric oxide release.^31^ The accumulation of symmetrical dimethylarginine in the HDL of patients with CKD also inhibits nitric oxide release, promoting a proinflammatory endothelial phenotype and impairing endothelial repair.^32^ Guanidinylation of triglyceride-rich lipoproteins such as apolipoprotein C3 also occurs in CKD. These guanidinylated lipoproteins then have proinflammatory properties and have been associated with cardiovascular events.^33,34^

### Accelerated Vascular Aging

It has been further hypothesized that the described toxic uremic milieu may disrupt normal vascular aging across the vasculature in a process referred to as inflammaging.^35^ Inflammaging is a prominent feature of the uremic phenotype, characterized by increased levels of inflammatory markers,^36^ proinflammatory cytokines,^37^ and reduced expression of Nrf2,^38^ contributing to premature vascular aging and an altered vascular smooth muscle cell pattern associated with accelerated arteriosclerosis.^39,40^ This abnormal myocardial and vascular remodeling in patients with CKD can contribute to cardiovascular complications, such as cardiomyopathy, ischemic heart disease, congestive heart failure, and stroke, as well as progression to kidney failure.^41^

CKD is linked to early cellular senescence,^42^ potentially through activation of the cyclin-dependent kinase inhibitors p16ink4a and p21cip1.^43,44^ A reduction in DNA damage repair mechanisms as individuals age can trigger a proinflammatory senescence-associated secretory phenotype. The senescence-associated secretory phenotype involves the release of proinflammatory cytokines like IL-1α, IL-1β, and IL-6, as well as ligands of the Wnt-β-catenin pathway and TGF-β.^42^

### Dysregulation of the Autonomic Nervous System

Dysregulation of the autonomic nervous system characterized by sympathetic overactivity and parasympathetic dysfunction commonly occurs in patients with CKD.^45^ This dysregulation contributes to systemic inflammation through various mechanisms. First, the sympathetic overactivity can potentiate inflammation through multiple pathways such as IFN-γ, IL-6, and IL-10.^46^ Second, impaired parasympathetic function in patients with CKD diminishes the anti-inflammatory effects mediated by the vagus nerve.^47^ The parasympathetic nervous system normally helps regulate inflammation by releasing acetylcholine, which inhibits the production of proinflammatory cytokines. Third, CKD-related autonomic dysfunction can impair baroreflex-mediated regulation of blood pressure, further exacerbating inflammation and promoting endothelial dysfunction, which are key factors in stroke pathogenesis.^48^

### Inflammatory Kidney Diseases

Inflammation may arise from autoimmune or inflammatory kidney disease etiologies as well as from the progression of kidney fibrosis.^14^ These inflammatory kidney diseases include antineutrophil cytoplasmic antibody-associated vasculitis, anti-glomerular basement membrane disease, lupus nephritis, IgA nephropathy, and other types of glomerulonephritis. Patients with proteinuric kidney diseases display mild inflammation associated with impaired endothelial function.^49^ Proteinuria contributes to CKD progression through associated adverse structural changes, including loss of selectivity in the glomerular barrier, glomerular enlargement, direct harm to tubule epithelial cells, and by promoting chronic inflammation.^50^ Tubulointerstitial fibrosis represents a final common pathway of different CKD etiologies and leads to irreversible deterioration of kidney function.^51^ In addition to driving atherosclerosis development through vascular aging, Nrf2 deficiency also associates with kidney fibrosis progression.^14^ IL-1β stimulates pericytes to promote kidney fibrosis^52^ and plays a role in activating T-helper 17 (TH17) cells within the kidney.^53^

### Exogenous Sources of Inflammation

Patients with CKD, and particularly those undergoing dialysis, are susceptible to frequent infections and thrombotic events, which also contribute to heightened inflammatory responses. These can manifest as catheter-related bloodstream infections, infections at access sites, thrombosis of arteriovenous fistulas and grafts, and episodes of peritonitis in patients undergoing peritoneal dialysis.^54^ Patients with CKD also often experience oral health issues,^55^ and the chronic inflammation seen in periodontal disease is linked to increased levels of systemic inflammatory markers as well as reduced overall survival of these patients.^56^

Dialysis-related factors also contribute to the inflammatory burden. Hemodialysis treatment itself acutely up-regulates transcription of proinflammatory cytokines.^57^ Numerous extracorporeal factors have been associated with inflammatory activation in dialysis patients, including impurities in dialysis water that may introduce endotoxins, the microbiological quality of the dialysate, as well as bioincompatible membranes and solutions used in the dialysis circuit.^58^

Furthermore, iron therapy, a cornerstone in the management of anemia among dialysis patients, has been associated with potential proinflammatory effects.^59^ While iron is essential for erythropoiesis, its administration can lead to oxidative stress. Intravenous iron preparations, commonly used in this population, may release labile iron, which can catalyze the formation of reactive oxygen species, triggering an inflammatory response.^60^ Iron overload, a consequence of prolonged or excessive iron therapy in this setting, is also a known risk factor for oxidative stress and inflammation.^61^ Iron therapy has been linked to elevated levels of proinflammatory cytokines, such as IL-6 and TNF-alpha.^62^

Finally, comorbidities like obesity and diabetes, which are highly prevalent in patients with CKD, can independently contribute to inflammation.^63^

## Time Course of Inflammatory Changes in CKD

The inflammatory changes in CKD progress in a time-dependent manner, correlating with the deterioration of kidney function. Even in early CKD (stages 1 and 2; eGFR >60 mL/min per 1.73 m²), subclinical signs of early inflammation can be detected, including small increases in inflammatory markers such as CRP (C-reactive protein) and IL-6.^64^ The renin-angiotensin-aldosterone system starts to become more active, which can lead to inflammation and fibrosis over time.^65^

In moderate (stage 3) CKD (eGFR, 30–59 mL/min per 1.73 m²), CRP, IL-6, and TNF-α levels begin to rise.^64^ There is an accumulation of uremic toxins like indoxyl sulfate and p-cresyl sulfate, which promote oxidative stress and inflammation.^20^ There is an increased production of reactive oxygen species, leading to cellular damage and further inflammation. Inflammatory processes start to cause fibrosis in kidney tissue, which exacerbates kidney function decline.^66^

In advanced (stage 4) CKD (eGFR, 15–29 mL/min per 1.73 m²), both innate and adaptive immune responses are heightened, leading to increased systemic inflammation.^9^ The imbalance between reactive oxygen species and antioxidants worsens, causing more oxidative damage, and fibrosis in the kidneys progresses, leading to further loss of kidney function and perpetuating the cycle of inflammation.

In CKD stage 5 (GFR <15 mL/min per 1.73 m^2^±requiring kidney replacement therapy), severe uremia contributes to systemic toxicity, and inflammatory markers are at their peak. CRP, IL-6, TNF-α, and other cytokines are markedly elevated, indicating widespread inflammation.^18^ Inflammation contributes to endothelial dysfunction and accelerated atherosclerosis with maximal risk of cardiovascular events in this stage.

However, while the cascade of inflammatory events in CKD involves common pathways such as renin-angiotensin-aldosterone system activation, oxidative stress, and immune activation, the specific triggers and extent of inflammation can vary significantly depending on the underlying cause of the disease. For example, diabetic nephropathy, hypertensive nephrosclerosis, glomerulonephritis, polycystic kidney disease, and obstructive nephropathy each have unique mechanisms that contribute to inflammation.^9,67–70^ In diabetic nephropathy, persistent hyperglycemia leads to the formation of advanced glycation end products, which induce oxidative stress and inflammation.^68^ In hypertensive nephropathy, hemodynamic stress causes direct damage to the glomeruli, leading to inflammatory responses.^68^ Immune complex deposition and complement system activation are the central mechanisms in glomerulonephritis.^9^ In polycystic kidney disease, mutations in polycystic kidney disease 1/2 lead to abnormal functioning of many key signaling pathways, which can result in abnormal proliferation, fibrosis, and inflammation that accompany cystogenesis.^69^ In obstructive uropathy, recurrent infections and tubule-interstitial damage promote an inflammatory state in the kidney.^70^

## Evidence Supportive of the Inflammatory Hypothesis for Stroke in CKD

**Figure 2.**
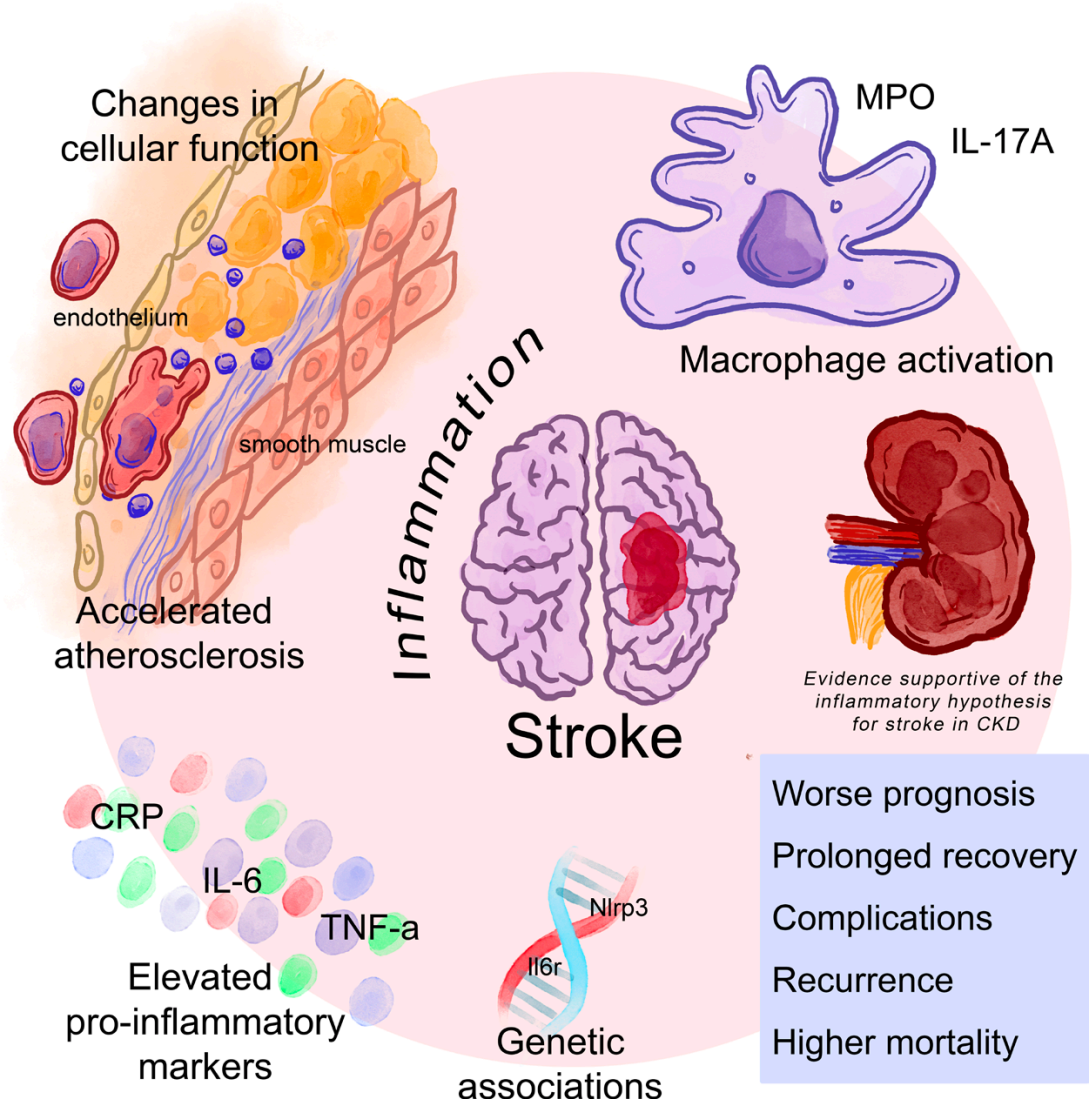
**Evidence supportive of the inflammatory hypothesis for stroke in chronic kidney disease (CKD).** CRP indicates C-reactive protein; IL, interleukin; MPO, myeloproteinase; and TNF, tumor necrosis factor.

The hypothesis that inflammation plays a significant role in the genesis and progression of stroke, especially in patients with CKD, is supported by a broad spectrum of studies, including basic science, clinical research, genetic association studies, and clinical trials (Figure 2).

In experimental models of stroke, CKD activates proinflammatory molecules in macrophages, such as myeloproteinase and IL-17A, both of which increase plaque burden and accelerate atherosclerosis in CKD mouse models.^71^ Reduced levels of active vitamin D in CKD-related mineral bone disease have been shown to increase adhesion molecules within endothelial cells and trigger a senescent state in smooth muscle cells. This senescent characteristic is commonly observed in the neointima of atherosclerotic plaques.^72^

Previous population-based cohort studies, including the ARIC (Atherosclerosis Risk in Communities), FHS (Framingham Heart Study), MESA (Multi-Ethnic Study of Atherosclerosis), and the CRIC (Chronic Renal Insufficiency Cohort) studies, have demonstrated that many circulating inflammatory biomarkers (including IL-6, TNF-α, high-sensitivity CRP [hsCRP], fibrinogen, and serum albumin) are increased in individuals with CKD, rising with declining kidney function in a graded relationship, and that they are independently predictive of incident cardiovascular events and death.^73–77^ Furthermore, these markers are also associated with a greater risk of kidney function decline.^77^

Inflammation may have a particularly strong role in atheroprogression in CKD, as demonstrated by the association between CKD, elevated inflammatory markers, carotid disease, and subsequent vascular events. In a community-based cohort study of over 3000 participants, it was found that kidney function significantly predicted higher carotid intima-media thickness, progression of subclinical atherosclerosis, and increased rates of cardiovascular events, independent of both traditional and nontraditional cardiovascular risk factors.^78^

In a study of plaque composition in patients with advanced carotid artery stenosis, patients with CKD had a higher prevalence of end-stage calcification, unstable and ruptured lesions, and lower collagen content in their plaques compared with those with normal kidney function.^79^ They also had much higher levels of relevant serum markers associated with inflammation, vascular calcification, and vessel wall degradation, including fibrinogen, parathyroid hormone, fetuin-A, and matrix metalloproteinase-7. Consistent with these findings, patients with CKD reported a higher prevalence of prior cerebrovascular events in the 6 months before carotid surgery compared with those with normal kidney function (84.0% versus 26.2%).

Epidemiological evidence for the potential interplay between CKD, inflammation, and stroke is further substantiated by genetic association studies. A meta-analysis comprising 82 studies of patients with coronary artery disease (CAD) identified a link between a specific variant in the IL-6R gene locus (rs2228145) and decreased systemic inflammation.^80^ Consequently, this variant was associated with a reduced risk of CAD, stroke, and myocardial infarction. In another large meta-analysis involving individual-participant data from a cohort of over 500 000 individuals, a highly prevalent intronic variant (rs10754555) within the NLRP3 (NLR family pyrin domain-containing 3) gene locus was found to be correlated with elevated NLRP3 inflammasome activity in human monocytes. This genetic variation was also associated with higher plasma levels of hsCRP, an increased likelihood of CAD, and higher cardiovascular mortality risk.^81^ Mendelian randomization analyses have also consistently demonstrated associations between the IL-6R locus and hsCRP levels.^82^ Phenome-wide association analyses have linked the IL-6R locus to various atherosclerotic phenotypes.^83^ Although genetic association studies investigating inflammation specifically in patients with CKD are limited, analyses based on data from the UK Biobank have revealed that genetic variations affecting IL-6 signaling have a more pronounced impact on CAD risk among individuals with CKD compared with those without CKD.^84^

As outlined in more detail in the next section, clinical trials based on anti-inflammatory interventions, such as statin therapy or anti-cytokine treatments, have also shown potential benefits in reducing the risk of cardiovascular events, including stroke, in patients with CKD.^85,86^

These findings collectively support the hypothesis that chronic inflammation in CKD is closely linked to the genesis and progression of atherosclerotic diseases such as stroke. Reducing inflammation and addressing its underlying causes may be potential strategies to mitigate stroke risk in patients with CKD.

## Inflammation in the Prognosis of Stroke

Inflammation in CKD can significantly impact all aspects of stroke prognosis, including severity, recovery, complications, vascular health, rehabilitation, and mortality (Figure 2). Inflammation is associated with more severe strokes in patients with CKD, which can lead to worse clinical outcomes. CKD-related inflammation may contribute to secondary brain injury, resulting in larger infarct volumes and more extensive neurological deficits.^87,88^ In mouse models of transient middle cerebral artery occlusion, the presence of CKD accentuated ischemic brain damage, resulting in increased apoptosis and neuronal loss in both the ischemic core and penumbra regions, ultimately impairing poststroke recovery.^89^ The acute phase of stroke recovery in CKD mice was characterized by heightened local inflammation, driven by increased activation of proinflammatory microglia/macrophages and reduced activation of reparatory ones.

Inflammation can also interfere with the recovery process after a stroke in patients with CKD. It may hinder neuroplasticity and the ability of the brain to compensate for damage, leading to slower and less complete functional recovery.^90^ It may contribute to a higher risk of nonvascular poststroke complications in these patients, such as infections, deep vein thrombosis, and cardiac events.^91,92^ These complications can further worsen the overall prognosis.

CKD-associated inflammation can accelerate atherosclerosis and endothelial dysfunction, making patients with CKD more susceptible to recurrent strokes and other vascular events.^93^ In a post hoc analysis involving 3020 patients with recent MRI-defined symptomatic lacunar infarction in the SPS3 trial (Secondary Prevention of Small Subcortical Strokes), individuals with CKD were found to have a 50% higher risk of recurrent stroke.^94^ It is possible that inflammation may also contribute to the higher short- and long-term mortality that is consistently observed in patients with CKD, regardless of stroke subtype, race, prevalence of vascular risk factors, or transplant status.^95–102^

## Novel and Established Anti-Inflammatory Therapies

**Figure 3.**
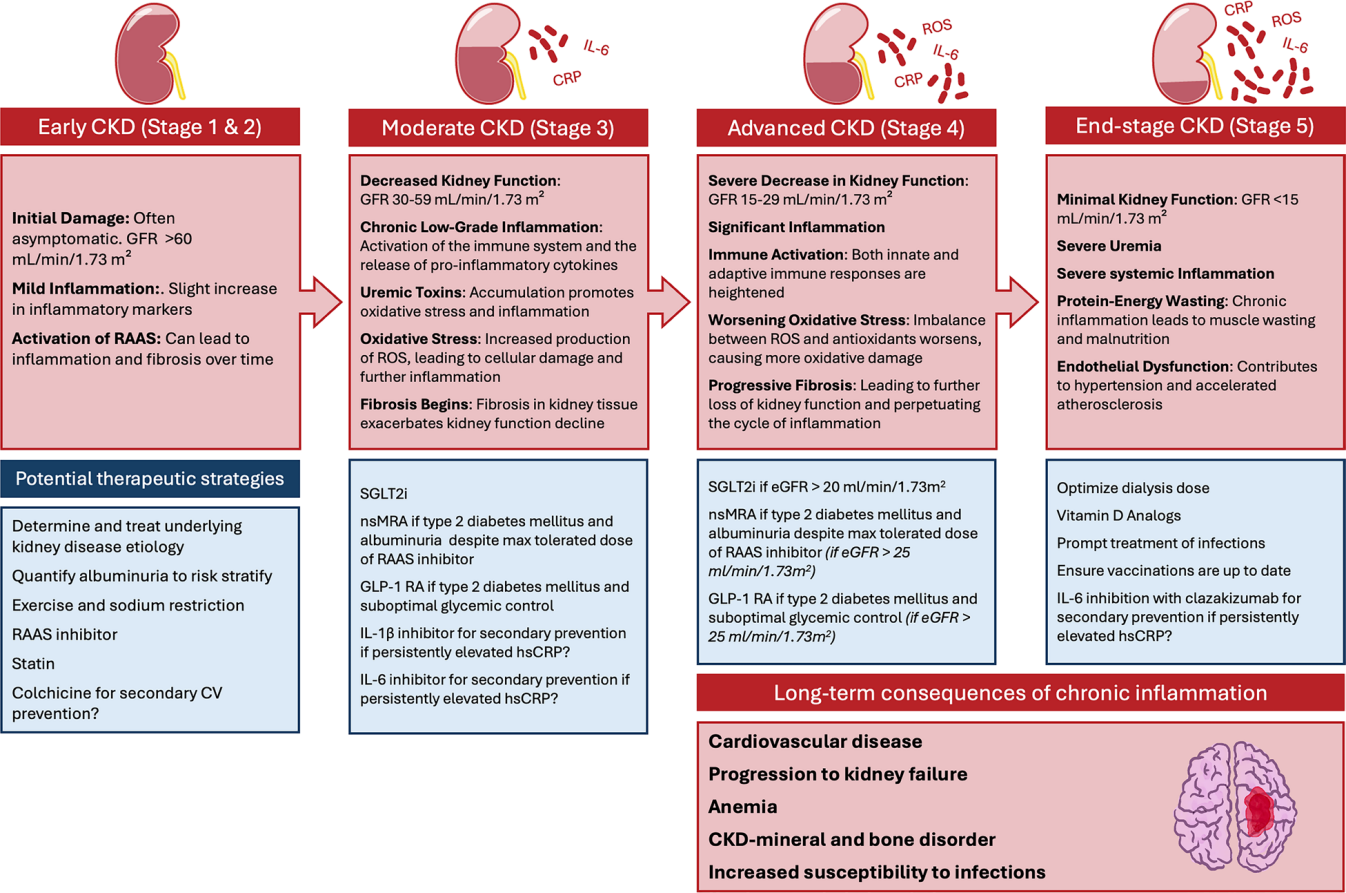
**Proposed inflammatory targets and therapeutic strategies based on chronic kidney disease (CKD) stage and progression.** CV indicates cardiovascular; GFR, glomerular filtration rate; GLP-1-RA, glucagon-like peptide-1 receptor agonist; IL, interleukin; nsMRA, nonsteroidal mineralocorticoid receptor antagonist; RAAS, renin-angiotensin-aldosterone system; ROS, reactive oxygen species; SGLT2i, sodium-glucose cotransporter 2 inhibitor; and T2DM, type 2 diabetes.

Inflammation and the activation of the innate immune system are recognized as significant contributors to both CKD and CVD.^19^ Despite the identification of various potential targets for anti-inflammatory treatments in CKD (Table 1), we currently lack definitive evidence that intervening in these pathways can prevent the development of CKD-related cardiovascular complications. We describe the current evidence for some of these potential anti-inflammatory treatment targets, and in Figure 3, we propose some putative therapeutic strategies based on CKD stage and progression.

**Table 1.**
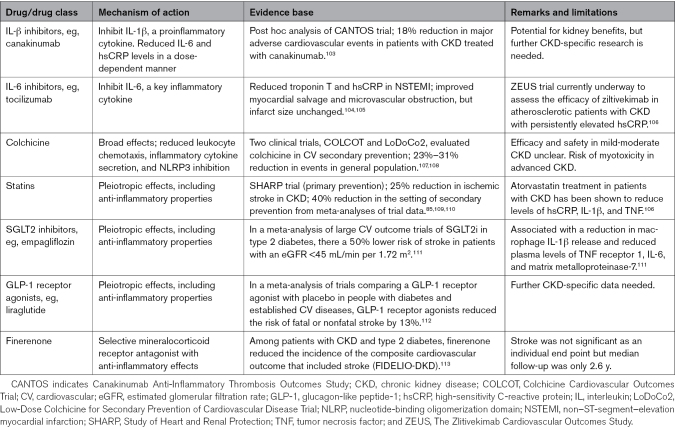
Potential Anti-Inflammatory Drugs for the Prevention of Stroke in Patients With CKD

### Inhibition of IL-1β

Small randomized controlled trials in patients with CKD suggest the potential benefits of inhibiting IL-1β. In one such study, patients with CKD were treated with the IL-1R antagonist anakinra for 4 weeks, resulting in a significant reduction in hsCRP and IL-6 levels by 53% and 40%, respectively, compared with a placebo group.^114^ Another trial using the IL-1 trap rilonacept observed reduced hsCRP levels and improved brachial artery flow-mediated dilation, a surrogate marker of endothelial function, in patients with CKD.^115^

The CANTOS study (Canakinumab Anti-Inflammatory Thrombosis Outcomes Study) enrolled 10 061 patients with a history of recent myocardial infarction and elevated hsCRP levels, randomly assigning them to receive either placebo or different doses of subcutaneous canakinumab, an IL-1β-targeting monoclonal antibody.^116^ Canakinumab significantly reduced IL-6 and hsCRP levels in a dose-dependent manner, with the higher doses (150 and 300 mg) decreasing the primary end point of nonfatal myocardial infarction, nonfatal stroke, or cardiovascular death by 15% compared with placebo. These benefits were maintained in a subgroup of 1875 patients with moderate CKD (eGFR, 30–60 mL/min per 1.73 m^2^) in whom an 18% reduction in major adverse cardiovascular events with canakinumab was reported.^103^

### Inhibition of IL-6

Following the success of IL-1β inhibition, IL-6 has also subsequently emerged as a potential atheroprotective target. Tocilizumab, an IL-6 receptor antibody, demonstrated possible benefit in 2 studies by lowering troponin T and hsCRP levels in non–ST-segment–elevation myocardial infarction and enhancing myocardial salvage while reducing microvascular obstruction.^104,105^ However, it did not impact infarct size in ST-segment–elevation myocardial infarction.

Ziltivekimab, a novel anti-IL-6 monoclonal antibody, is in development to prevent atherosclerosis in moderate-to-severe patients with CKD. In the RESCUE trial (Trial to Evaluate Reduction in Inflammation in Patients With Advanced Chronic Renal Disease Utilizing Antibody Mediated IL-6 Inhibition), it substantially reduced hsCRP levels (up to 92%), along with other relevant markers like fibrinogen, serum amyloid A, and lipoprotein(a).^86^ Ziltivekimab demonstrated good tolerability and led to the ZEUS trial (Zlitivekimab Cardiovascular Outcomes Study), which is currently underway and recruiting specifically atherosclerotic patients with CKD with persistently elevated hsCRP, aiming to assess atherosclerotic event reduction and potential kidney function improvement.^106^

Recently, a randomized phase 2b trial assessed clazakizumab, a monoclonal antibody targeting IL-6, in adults with CVD or diabetes on maintenance dialysis with elevated hsCRP levels.^117^ Clazakizumab significantly reduced hsCRP levels by 86%, 90%, and 92% relative to placebo in patients randomized to 2.5, 5, or 10 mg doses. Achieved secondary end points included reductions in serum fibrinogen, amyloid A, secretory phospholipase A2, and lipoprotein(a), and an increase in serum albumin. The study concluded that clazakizumab effectively reduces inflammatory biomarkers associated with CVD in maintenance dialysis patients. Further studies are needed to determine whether a reduction in these surrogate markers would later correspond with a reduction in cardiovascular event rates.

### Colchicine

Unlike targeted cytokine inhibitors like canakinumab and ziltivekimab, colchicine, traditionally used to treat gout, is a nonspecific anti-inflammatory agent with broad effects, including reduced leukocyte chemotaxis, inflammatory cytokine secretion, and NLRP3 inhibition.^118^

Two trials, COLCOT (Colchicine Cardiovascular Outcomes Trial) and LoDoCo2 (Low-Dose Colchicine for Secondary Prevention of Cardiovascular Disease Trial), evaluated colchicine in CVD secondary prevention in the general population.^107,108^ COLCOT included 4745 patients within 30 days after myocardial infarction and showed a 23% reduction in the primary cardiovascular end point with colchicine versus placebo, while LoDoCo2 recruited 5522 patients who had stable CAD for at least 6 months and demonstrated a 31% risk reduction in CV events. A 2021 meta-analysis further supported colchicine’s efficacy in reducing major adverse cardiovascular events, including myocardial infarction, stroke, and coronary revascularization.^119^ However, there was a trend toward higher noncardiovascular mortality, the cause of which was unclear. In the recently published CONVINCE trial (Colchicine for Prevention of Vascular Inflammation in Non-Cardioembolic Stroke), patients treated with long-term colchicine had numerically fewer recurrent stroke and coronary events compared with those on guideline-based therapy only, but the difference was not statistically significant in the intention-to-treat analysis.^120^

Notably, these trials excluded patients with advanced CKD. Colchicine is relatively contraindicated in moderate-to-severe CKD due to a higher risk of myotoxicity, even with short-term use.^121^ Impaired kidney function can lead to decreased clearance of colchicine and increased risk of adverse effects, including diarrhea and other gastrointestinal symptoms.^122^ Due to the greater potential for toxicity, patients with CKD receiving colchicine therapy may require closer monitoring of kidney function and hematologic parameters. Dosing adjustments may be necessary to minimize the risk of adverse events while ensuring therapeutic efficacy.

### Other Therapies With Anti-Inflammatory Effects

Beyond innovative strategies targeting innate immunity, several well-established therapies may have pleiotropic anti-inflammatory effects that are promising for reducing the cardiovascular burden in CKD. The efficacy of statin therapy in patients with CKD has been well established from the SHARP trial (Study of Heart and Renal Protection)^85^ and from subsequent meta-analyses in which there was a ≈40% reduction in the risk of stroke in patients with CKD.^109,110^ Notably, atorvastatin treatment in patients with CKD has been shown to reduce levels of hsCRP, IL-1β, and TNF, with these reductions not solely attributable to cholesterol lowering, suggesting statins possess additional antioxidant and anti-inflammatory properties.^123^

SGLT2 (sodium-glucose cotransporter 2) inhibitors, known for their nephroprotective and cardiovascular benefits in patients with CKD, irrespective of diabetes status, may also possess anti-inflammatory effects.^124^ For instance, in patients with type 2 diabetes, empagliflozin has demonstrated a reduction in macrophage IL-1β release, implying inhibition of the NLRP3 inflammasome.^125^ Similarly, canagliflozin has been associated with reduced plasma levels of TNF receptor 1, IL-6, and matrix metalloproteinase-7.^126^ In a meta-analysis of large cardiovascular outcome trials of SGLT2 inhibitors in type 2 diabetes, there was evidence of a 50% lower risk of stroke specifically in patients with an eGFR <45 mL/min per 1.72 m^2^.^111^

Additionally, 2 other pillars of cardioprotective care, GLP-1 (glucagon-like peptide-1) receptor agonists and finerenone (a selective mineralocorticoid receptor antagonist) have shown various anti-inflammatory effects while proving effective in slowing CKD progression and reducing cardiovascular events, as demonstrated in the FIDELIO-DKD trial (Finerenone in Reducing Kidney Failure and Disease Progression in Diabetic Kidney Disease).^127,128^

Lipid-lowering agents such as bempedoic acid and ezetimibe have also demonstrated favorable effects on hsCRP levels, particularly when used in combination with statin therapy. The pursuit of combining potent lipid-lowering agents with robust anti-inflammatory inhibitors represents a promising avenue for future drug development, potentially involving combinations of agents or bi-specific monoclonal antibodies.^19^

## Future Research Directions

Research priorities for anti-inflammatory therapies for stroke in patients with CKD, as outlined in Table 2, should include dedicated clinical trials, combination approaches, longer-term safety data, mechanistic and genetic studies, among others.

**Table 2.**
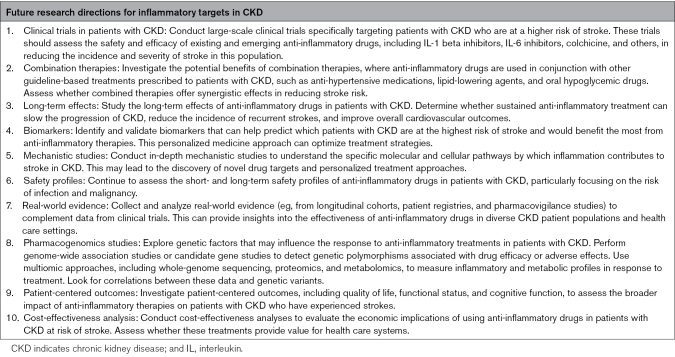
Research Priorities for Investigating the Utility of Anti-Inflammatory Therapies for Stroke Prevention and Treatment in Patients With CKD

The innate immune system offers multiple potential therapeutic targets due to its intricate pathways leading to systemic inflammation. In animal models of CVD, targeting the NLRP3 inflammasome has shown promise in reducing atherosclerosis and myocardial damage.^129^ However, translating these findings to humans has proven challenging, as exemplified by the halted phase II trial of MCC950 in rheumatoid arthritis patients due to hepatotoxicity.^130^

JAK2, integral to IL-6R signaling and linked to atherosclerosis, is being explored as a target using JAK inhibitors, primarily in myeloproliferative diseases.^131^ Ruxolitinib, a JAK1 and JAK2 inhibitor, demonstrated mixed effects in murine atherosclerosis models, reducing lesion size but promoting unstable plaque composition.^132^

Pentraxins are a family of proteins involved in the innate immune response and inflammation. Agents targeting pentraxins, such as PTX3 inhibitors, are being explored for their potential to modulate inflammation and reduce cardiovascular risk in patients with CKD.^133^ Therapeutic targeting of Damage-Associated Molecular Patterns associated with CKD also appears to be a promising strategy to reduce chronic vascular inflammation and cardiovascular risk.^134^

## Conclusions

The relationship between CKD, inflammation, and stroke is clearly complex and multifactorial. While inflammation may be a significant contributor to stroke risk, evolution and outcomes in patients with CKD, other factors such as age and multimorbidity also play key roles that are harder to mitigate. Managing inflammation and addressing its underlying causes in patients with CKD may be a potential strategy for reducing stroke risk in this population.

## Article Information

### Acknowledgments

Drs Kelly and Rothwell participated in the conceptualization of the article. Dr Kelly is responsible for the writing—original draft preparation—of the article. E.M. Kelleher participated in the visualization of the article. Dr Rothwell provided supervision of the article. All authors are responsible for the writing (review and editing), and have read and agreed to the published version of the article.

### Sources of Funding

Dr Kelly is supported with funding from Guarantors of Brain and from the Global Brain Health Institute, Alzheimer’s Association, and Alzheimer’s Society (GBHI ALZ UK-22-868940).

### Disclosures

Dr Rothwell has in the past provided consultancy services for Abbott Vascular, Bayer, Bristol-Myers Squibb, and Sanofi US Services. The other authors report no conflicts.
